# Sequential Patterns and Transition Timelines of Chronic Disease Comorbidities in Obesity: Evidence from the ELSA database

**DOI:** 10.21203/rs.3.rs-7020515/v1

**Published:** 2025-08-13

**Authors:** Bingsong Zhang, Zuyi Zhao, Haixin Feng, Siran Li, Yalin Kuang, Zhirong Zeng

**Affiliations:** Guangdong Medical University; Beijing Normal University, Ministry of Education; Guangdong Medical University; Guangdong Medical University; Guangdong Medical University; Guangdong Medical University

**Keywords:** obesity, multimorbidity, disease progression

## Abstract

**Objective:**

To characterize the sequential patterns and transition timelines of chronic disease comorbidities in population with obesity.

**Methods:**

We analyzed population with obese from the English Longitudinal Study of Ageing, including 22,355 independent participants for using association rule mining (ARM) to identify comorbidity patterns and 92,092 person-observations to analyze disease progression pathways and transition probability by multi-stage Markov chain (MMC). Health burden was compared between different onset disease.

**Results:**

ARM identified cardiovascular (CVD), metabolic (MTD), and skeletal-muscular disease (SMD) as the most prevalent disease trio. MMC revealed 40% of obese individual will develop a chronic disease within 5 years, and nearly 30% with MTD or CVD will develop to the trio within 10 years. Progression times to the trio differed significantly based on initial disease type (*p* < 0.003), with MTD onset being the fastest progression (3.89 years). SMD onset was associated with the most adverse health burden profile, including the highest depression rate (6.3%), poorest sleep quality (77.0%), and substantial work limitations (74.0%).

**Conclusions:**

These findings establish quantifiable transition probabilities and timelines for chronic disease progression, emphasizing the important role of onset disease and contributing empirical evidence for the sequential nature of multimorbidity development.

## 1. Introduction

Obesity has emerged as one of the most pressing public health challenges of the 21st century, affecting over 650 million adults worldwide and representing a nearly threefold increase in prevalence since 1975 [1]. In developed nations, including the United Kingdom and United States, more than one-third of the adult population is classified as obese (BMI ≥ 30 kg/m^2^), with projections indicating continued growth in prevalence across all demographic groups [2, 3]. This epidemic extends far beyond issues of body weight, as obesity serves as a catalyst for numerous chronic diseases that collectively contribute to substantial morbidity, mortality, and healthcare expenditure [4].

The pathophysiological consequences of obesity are multifaceted and interconnected. Excess adipose tissue, particularly visceral fat, functions as an active endocrine organ that secretes inflammatory cytokines, adipokines, and other bioactive molecules that disrupt metabolic homeostasis [5]. These mechanisms contribute to insulin resistance, dyslipidemia, chronic inflammation, and endothelial dysfunction—processes that predispose individuals to cardiovascular disease, type 2 diabetes, and metabolic syndrome [6, 7]. Additionally, the mechanical burden of excess weight places strain on musculoskeletal structures, contributing to arthritis and other degenerative joint conditions [8]. The clustering of chronic diseases in obese individuals, termed multimorbidity, represents a significant clinical challenge. Studies consistently demonstrate that obese individuals are at substantially higher risk for developing multiple chronic conditions compared to their normal-weight counterparts [9, 10]. This multimorbidity burden is associated with accelerated functional decline, reduced quality of life, increased healthcare utilization, and premature mortality [11, 12].

While the association between obesity and individual chronic diseases is well-established, the temporal sequence and progression patterns of comorbidity development remain poorly understood. Traditional cross-sectional studies provide snapshots of disease co-occurrence but fail to capture the dynamic nature of disease evolution over time [13]. Understanding these sequential patterns is crucial for several reasons: first, it can inform risk stratification and early intervention strategies; second, it may reveal critical windows for preventive interventions; and third, it can guide resource allocation and care coordination for high-risk populations [14, 15]. The concept of disease trajectories—the pathways through which individuals progress from health to single disease states and subsequently to multimorbidity—has gained increasing attention in epidemiological research [16]. However, most existing studies have focused on general adult populations or specific age groups, with limited investigation of these patterns specifically within obese populations where multimorbidity burden is disproportionately high [17, 18].

Despite the recognized importance of understanding disease progression in obesity, several critical knowledge gaps persist that limit our ability to develop effective interventions and optimize clinical care. While individual disease associations with obesity are well-documented, there remains limited evidence regarding the most common sequences of chronic disease development specifically in obese populations [19, 20]. The timelines for transitions between different disease states are also poorly characterized, hindering the development of evidence-based screening protocols and optimal intervention timing [21]. Perhaps most importantly, it remains unclear whether the type of initial comorbidity influences subsequent disease development patterns and associated health burden—a question with significant implications for prioritizing early intervention targets [22, 23]. These interconnected knowledge gaps are particularly problematic given that obese individuals often present with complex, multifaceted health conditions that require coordinated management approaches, yet our understanding of how these conditions evolve over time remains fragmented [24].

The primary objective of this study is to characterize the sequential patterns and transition timelines of chronic disease comorbidities in obese individuals using data from the English Longitudinal Study of Ageing (ELSA) [25]. Specifically, we aim to: (1) identify the most prevalent patterns of chronic disease co-occurrence among obese adults using association rule mining; (2) model the progression pathways and transition probabilities between different disease states using multi-state Markov modeling; (3) estimate the timelines for disease progression from single to multiple comorbidities; and (4) assess whether different initial disease patterns are associated with varying levels of disease burden. By integrating cross-sectional pattern recognition with longitudinal transition modeling, this study seeks to provide comprehensive insights into the natural history of multimorbidity development in obesity, thereby informing evidence-based strategies for prevention, early intervention, and clinical management.

## 2. Materials

### 2.1. Data Source

This study utilized data from the ELSA, a prospective cohort study of adults aged 50 years and older living in England. ELSA was initiated in 2002 with participants drawn from the Health Survey for England (HSE), and follow-up assessments were conducted biennially. For each wave, the ELSA study includes measurements of health status, socioeconomic circumstances, and well-being. Data collection involves face-to-face interviews, self-completion questionnaires, and nurse visits (every four years) for anthropometric measurements and biomarker collection. All participants provided written informed consent, and ethical approval was granted by the London Multicenter Research (MREC/01/2/91).

### 2.2. Study Population

**Figure 1:**
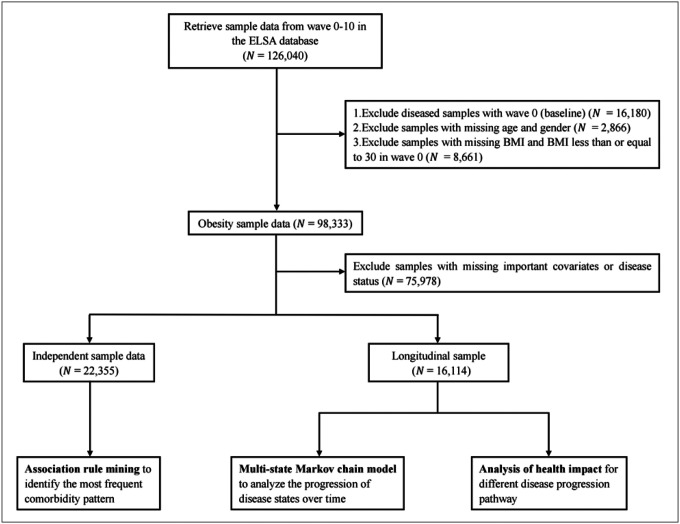
Sample Selection Process and Analytical Framework

In this study, we used data from Wave 0 (1998–2001, baseline HSE data) through Wave 10 (2020–2021) and employed a two-part approach: a cross-sectional analysis to identify common comorbidity patterns in obese individuals and a longitudinal analysis to investigate the dynamic transition between different disease states. The sample selection process and analysis plan were illustrated in Fig. 1.

For the independent sample, we extracted unique samples or retained the last available survey data for those with repeated measurements. This approach resulted in 22,355 participants for analysis to find the comorbidity patterns. For the longitudinal sample, we selected participants with at least two survey measurements. By excluding participants with missing data on key variables, we retained 92,092 person-observations from 16,114 participants to estimation the transition probabilities. We excluded individuals with missing anthropometric data at baseline, were non-obese, or had information on chronic disease status. For the longitudinal analysis, complete follow-up data on transitions between disease states was required.

### 2.3. Obesity

Participants were classified as obese if their body mass index (BMI) ≥ 30 kg/m^2^, aligning with the World Health Organization’s (WHO) definition and the International Diabetes Federation guidelines for obesity.

### 2.4. Chronic Disease

Chronic disease information in ELSA was collected using standardized questionnaires that have been validated and used consistently across all ELSA waves [25]. The questionnaires assess self-reported physician diagnoses during structured interviews, following established protocols described in the ELSA methodology documentation [25]. These instruments were not developed for this study but represent standard ELSA data collection procedures. Participants were asked whether they had ever been diagnosed with specific conditions, whether they still had the condition, and if they were taking medication or receiving treatment for it. A disease was considered present if any of these questions answered affirmatively.

Based on the disease classification frameworks of the WHO’s International Classification of Diseases (ICD-11), we categorized the specific conditions reported in ELSA into seven major disease groups. Cardiovascular system diseases (CVD) included heart disease, angina, hypertension, varicose veins, thrombosis, heart failure, heart murmur, and arrhythmia. Metabolic system disease (MTD) included high cholesterol, high blood glucose, diabetes, and abnormal endocrine metabolism. Respiratory system disease (RPD) includes bronchitis, emphysema, asthma, hay fever, lung diseases, and respiratory ailments. Skeletal-muscular system diseases (SMD) included arthritis, rheumatism, fibromyalgia, slipped disc, gout, and problems with back, spine, neck bones, joints, and muscles. Neurological system diseases (NRD) covered epilepsy, migraine, stroke, cerebral hemorrhage, Parkinson’s disease, Alzheimer’s disease, dementia, multiple sclerosis, and motor neuron disease. Mental disorders (MD) included various mental illnesses and psychiatric disorders. Cancer (CC) encompassed all types of cancers.

### 2.5. Health Burden Measures

To assess the impact of chronic disease on participants’ overall well-being and functioning, we defined several health burden measures. These included self-reported health status (HRS version: excellent, very good, good, fair, poor), presence of health problems or disabilities that limited paid work (yes/no), sleep quality rating (excellent, very good, good, fair, poor), and presence of depression (yes/no).

### 2.6. Statistical Analysis

#### Descriptive Analysis.

For the independent sample, we conducted descriptive analyses to characterize the study population. Participants were stratified by their comorbidity status (no chronic disease, one chronic disease, two chronic diseases, and three or more chronic diseases), and demographic and system diseases were compared across these groups. Continuous variables were presented as means and standard deviations, while categorical variables were reported as frequencies and percentages.

#### Association Rule Mining (ARM).

To identify the most frequent comorbidity patterns among obese individuals, we employed Apriori algorithm-based ARM on the independent sample. This data mining technique was used to discover meaningful associations between different chronic diseases and identify frequently co-occurring disease combinations.

The analysis was conducted with a minimum support threshold of 0.005 and a confidence level of 0.07 to ensure statistical relevance of identified patterns. To examine demographic variation in comorbidity patterns, we stratified the association rule mining by age groups (19–37, 38–56, 57–75, and 76–94 years) and by gender. From this analysis, we identified the most prevalent three-disease combination (trio) for subsequent longitudinal modeling. The “*arules*” package in R version 4.4.3 was used for this analysis.

#### Multi-state Markov Chain Model (MMC).

**Figure 2:**
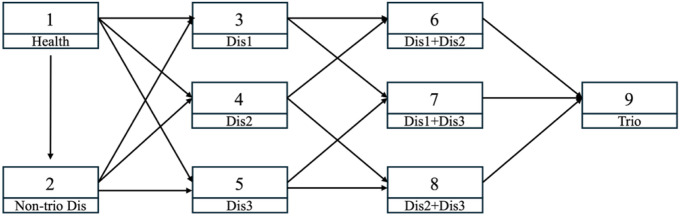
State Transition Diagram for Chronic Disease Progression in Obese Individuals Note: State 1 represents the healthy state (no chronic diseases); State 2 represents non-trio single disease; States 3–5 represent single disease states; States 6–8 represent dual comorbidity states; and State 9 represents the triple comorbidity state. Arrows indicate clinically plausible transition pathways.

Based on the results from ARM, we constructed a multi-state Markov chain model to analyze the progression of disease states over time. Nine distinct health states were defined: State 1: No chronic disease (healthy state); State 2: Disease present, but not part of the identified trio; State 3: First disease of the identified trio only; State 4: Second disease of the identified trio only; State 5: Third disease of the identified trio only; State 6: First and second diseases (dual comorbidity); State 7: First and third diseases (dual comorbidity); State 8: Second and third diseases (dual comorbidity); State 9: All three diseases (triple comorbidity). We implemented a discrete-time Markov model to estimate the transition rates between these health states using the longitudinal sample. The allowable transitions between health states were structured according to clinically plausible disease progression pathways, as illustrated in Fig. 2. This directed graph represents the possible transitions from the healthy state to various comorbidity states, with arrows indicating permitted progression routes. For example, from the healthy state (State 1), individuals could transition to State 2 (non-trio disease) or to single-disease states of the identified trio (States 3, 4, or 5). From single-disease states, transitions were only allowed to dual-comorbidity states that included the initial disease. The triple comorbidity state (State 9) was defined as an absorbing state.

From the fitted model, we estimated transition intensities representing the instantaneous risk of moving between states, transition probabilities over specified time intervals. We calculated the time to develop triple comorbidity from different starting states and tested the statistical significance from the longitudinal sample to assess whether the type of onset disease affected the subsequent comorbidity development timeline.

#### Analysis of Health Burden.

To evaluate whether different onset diseases are associated with varying levels of health outcome, we analyzed the health burden measures across different disease progression pathways. For each impact measure, we computed the prevalence or mean score for participants who followed different transition sequences. Comparisons between different pathways were performed using chi-square tests for categorical burden measures and ANOVA for continuous measures.

All statistical analyses were performed using R version 4.4.3. The “*msm*” package was used for multi-state modeling. Statistical significance was set at *p* < 0.05 for all analyses.

## 3. Results

### 3.1. Association rule mining from the independent sample

Table 1 presents the baseline characteristics of the independent sample stratified by the number of chronic diseases. Participants with multiple comorbidities were older and showed progressively higher prevalence of each disease category. Cardiovascular diseases were most prevalent across all comorbidity groups, affecting 32.29% of those with one disease and 79.46% of those with three or more disease.

ARM analysis identified CVD, MTD, and SMD as the most prevalent disease trio among obese individuals (Tables 2 and 3). Gender-stratified analysis revealed distinct comorbidity patterns: CVD-MTD combinations predominated in males, while CVD-SMD combinations were more common in females. The triple comorbidity of CVD-MTD-SMD showed similar prevalence across genders. Age-stratified analysis demonstrated progressive shifts in comorbidity patterns across the lifespan. In the youngest group (19–37 years), respiratory conditions were most prevalent (50%), while CVD emerged as the predominant single condition in middle age (30.30% in 38–56 years). The CVD-MTD-SMD triple comorbidity was especially common in the oldest age group (76–94 years, 30.99%). Based on its high prevalence and strong association metrics across age groups, this trio was selected for subsequent longitudinal modeling. CVD, MTD, and SMD were set as state 3, 4, and 5 in Fig. 2, respectively.

### 3.2. Multi-state Markov Model from Longitudinal Sample

The longitudinal sample comprised 92,092 person-observations from 16,114 participants with a mean follow-up of 10.68 years. Table 4 shows the evolution of participant characteristics from first to last follow-up. The proportion with no comorbid diseases decreased substantially from 32.76–12.47%, while those with three or more diseases increased from 10.90–41.90%, demonstrating the progressive accumulation of comorbidities over time. Notably, the prevalence of metabolic diseases nearly doubled (11.59–20.68%), while skeletal-muscular diseases remained relatively stable (25.03–20.43%).

**Figure 3:**
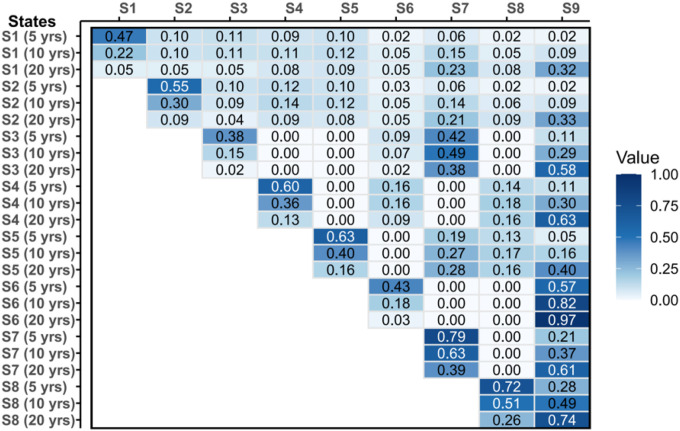
Estimated 5-, 10-, and 20-Year Transition Probability Matrices Between Disease States in Obese Participants Based on Markov Chain Modeling Note: S1: health state; S2: non-trio single disease; S3: CVD; S4: MTD; S5: SMD; S6: CVD+MTD; S7: MTD+SMD; S8: CVD+SMD. S9: CVD+MTD+SMD.

Figure 3 presents the estimated transition probability matrices of disease states for 5-, 10-, and 20-year periods in obese individuals. The matrices reveal that disease development follows a sequential pattern, with direct transitions from healthy state to complex multimorbidity remaining uncommon at 5 years but becoming increasingly probable over longer periods. Figure 3 shows that 40% of obese 60-year-olds with no other chronic disease will develop a chronic disease within 5 years, and nearly 30% of obese people with MTD or CVD will develop to the trio within 10 years. Notably, transitions involving metabolic disease components consistently show higher probabilities compared to other pathways, supporting the role of metabolic dysfunction as a key driver of multimorbidity progression and providing quantitative evidence for accelerating disease accumulation over time.

Statistical analysis of transition times revealed significant differences in disease development patterns. Individuals starting from state 2 developed CVD, MTD, and SMD significantly faster than those starting from state 1, with all comparisons yielding *p* < 0.001, indicating that the presence of any chronic disease accelerates the development of the core obesity -related comorbidity trio.

**Figure 4:**
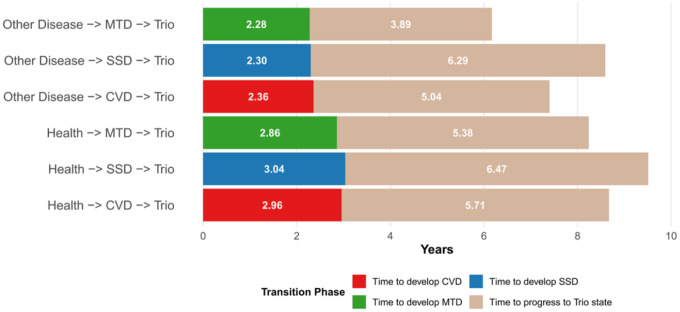
Transition times from Health and Single Disease States to Combined CVD-SMD-MTD (Trio) States

Figure 4 illustrates the transition times from healthy and single disease states to the combined CVD-MTD-SMD trio states. Critically, the time to develop triple comorbidity (state 9) differed significantly based on the type of initial chronic disease (*p* < 0.003). Bonferroni-adjusted pairwise comparisons revealed that progression from MTD onset differed significantly from both CVD onset (*p* = 0.016) and SMD onset (*p* = 0.014). However, no significant difference was observed between CVD and SMD onset pathways (*p* = 0.46). These results demonstrate that metabolic disease serve as particularly rapid catalysts for multimorbidity development, while cardiovascular and skeletal-muscular disease show similar, slower progression patterns to triple comorbidity.

### 3.3. Health Burden Analysis

**Figure 5:**
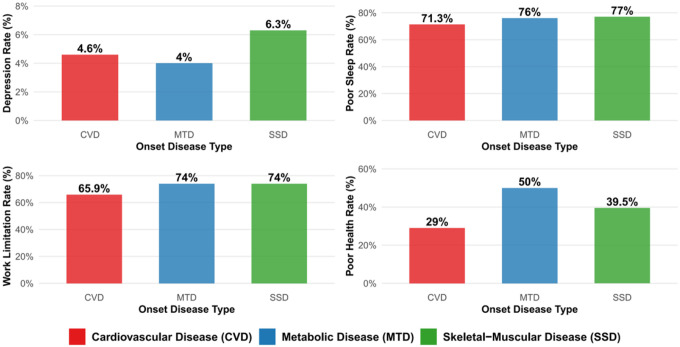
Health Burden by Onset Chronic Disease Type in Obese Population

Analysis of health burden measures revealed significant differences based on the type of initial chronic disease onset (Fig. 5). Individuals with SMD as their first chronic condition demonstrated the most adverse impact profile across multiple health domains. Specifically, SMD onset was associated with the highest depression rate compared to CVD and MTD onset (*p* = 0.048). Poor sleep quality was prevalent across all onset types but reached its peak with SMD onset (*p* = 0.005). Work limitations showed that both MTD and SMD onset associated with high rates, substantially higher than CVD onset (*p* < 0.001). For self-rated poor health status, MTD onset showed the highest prevalence compared to SMD and CVD (*p* < 0.001). These findings demonstrate that while all three disease types impose substantial burden, SMD onset is associated with the most consistent elevation across multiple health domains, suggesting its role as a particularly problematic “gateway” condition in obese individuals.

## 4. Discussion

In this study, we demonstrate that chronic disease development in obesity follows predictable sequential patterns, with CVD-MTD-SMD representing the most common comorbidity cluster. The identification of this trio aligns with established pathophysiological mechanisms linking obesity to these conditions through adipose tissue dysfunction, chronic inflammation, and mechanical stress [5, 6]. Gender differences in comorbidity patterns likely reflect hormonal factors, lifestyle patterns, and occupational exposures, while age-stratified patterns underscore the cumulative nature of disease burden in aging obese populations. Multi-state Markov modeling revealed that disease development follows predictable rather than random patterns, with quantifiable probabilities for progression between disease states. This temporal modeling approach captures the dynamic nature of multimorbidity development and provides clinically relevant estimates for risk stratification and intervention timing. Importantly, progression times to triple comorbidity differed significantly based on initial disease type, with metabolic disease onset demonstrating the fastest progression, while cardiovascular and skeletal-muscular diseases showed similar, slower progression patterns. Despite this rapid progression timeline, the finding that skeletal-muscular system disease as an initial condition leads to particularly poor health outcomes across multiple domains. Musculoskeletal conditions can severely limit physical activity and mobility, creating cycles that accelerate weight gain and worsen metabolic dysfunction [6, 8]. Chronic pain associated with these conditions contributes to sleep disturbances, mood disorders, and reduced quality of life [26, 27].

The dual-method approach combining ARM with MMC modeling captured both independent disease clustering and longitudinal evolution patterns. ELSA data provided sufficient temporal resolution over 20 years to capture disease transitions accurately, while stratification by gender and age groups revealed important heterogeneity. These findings have important clinical implications. Common comorbidity patterns can inform integrated care models that address multiple related conditions simultaneously. The particularly adverse impact of SMD as an initial condition suggests that musculoskeletal health should be prioritized in obesity management through aggressive screening, earlier specialist referral, and targeted exercise programs. Transition probability estimates could be incorporated into clinical decision support tools for risk assessment and resource allocation in primary care settings.

Several limitations should be acknowledged. Self-reported physician diagnoses may be subject to recall bias, though validation studies show good agreement for major chronic conditions. We focused on BMI-defined obesity without distinguishing adipose tissue distributions or metabolic health status. The biennial data collection schedule of ELSA may introduce temporal bias, as the exact timing of disease onset cannot be observed between waves, potentially leading to overestimation of disease development times since participants must wait until the next survey wave for disease reporting. This suggests that actual progression times may be shorter than our calculated estimates. Future studies might incorporate measures of central adiposity or metabolic syndrome components for refined risk stratification. We did not examine specific interventions that might modify progression pathways.

This study provides evidence that skeletal-muscular system disease as an initial condition leads to particularly poor outcomes in obese individuals, while metabolic disease onset demonstrates the fastest progression to multimorbidity. The methodological framework offers a comprehensive approach to understanding multimorbidity development that could be applied to other populations and disease contexts. Our findings establish quantifiable transition probabilities and timelines for chronic disease progression in obesity, contributing empirical evidence for the sequential nature of multimorbidity development and the differential impact of initial disease types on subsequent health outcomes.

## Figures and Tables

**Table 1:** Baseline Characteristics of Obese Participants Stratified by Number of System-Based Disease in the Cross-sectional Sample (*N* = 22,355)

Variables	Levels	With no disease	With one disease	With two diseases	With three or more diseases
		(*N* = 4,207)	(*N* = 4,940)	(*N* = 5,329)	(*N* = 7,879)
Age		59.91	65.78	69.61	72.73
		(10.75)	(11.43)	(11.08)	(10.01)
BMI		29.47	27.40	27.41	27.60
		(6.06)	(6.57)	(7.28)	(8.62)
Gender	Male	1973	2313	2544	3364
		(46.90)	(46.82)	(47.74)	(42.70)
	Female	2234	2627	2785	4515
		(53.10)	(53.18)	(52.26)	(57.30)
Race	White	3693	4360	4824	7314
		(93.78)	(94.70)	(95.09)	(95.20)
	Non-white	245	244	249	369
		(6.22)	(5.30)	(4.91)	(4.80)
Cardiovascular system diseases		-	1596	3293	3374
			(32.29)	(61.72)	(79.46)
Metabolic system disease		-	689	2230	2734
			(13.94)	(41.8)	(64.39)
Respiratory system disease		-	459	805	1022
			(9.29)	(15.09)	(24.07)
Skeletal-muscular system diseases		-	1090	2124	2685
			(22.06)	(39.81)	(63.24)
Neurological system diseases		-	608	1179	1580
			(12.30)	(22.10)	(37.21)
Mental disorders		-	260	547	710
			(5.26)	(10.25)	(16.72)
Cancer		-	240	492	633
			(4.86)	(9.22)	(14.91)

**Table 2:** Most Frequent System Disease Combinations in Obese Individuals Stratified by Gender

Comorbidity	Order	Overall			Female			Male		
		System Disease	%	lift	System Disease	%	lift	System Disease	%	lift
Singleton	1	CVD	32.29	1.04	CVD	28.83	1.04	CVD	36.23	1.04
	2	SMD	22.06	1.07	SMD	26.06	1.06	SMD	17.51	1.08
	3	MTD	13.94	1.13	NRD	14.30	0.93	MTD	16.95	1.13
	4	RPD	13.94	0.96	MTD	11.30	1.14	RPD	10.81	0.88
	5	NRD	12.30	0.92	RPD	7.95	1.02	NRD	10.03	0.90
	6	MD	5.26	0.96	MD	6.77	0.97	CC	4.93	1.01
	7	CC	4.86	1.02	CC	4.79	1.04	MD	3.55	0.92
Pair	1	CVD, MTD	25.21	1.58	CVD, SMD	21.51	16.68	CVD, MTD	33.35	1.53
	2	CVD, SMD	17.94	1.38	CVD, MTD	17.78	7.01	CVD, SMD	14.02	1.39
	3	CVD, NRD	7.18	1.28	MTD, SMD	8.00	4.28	CVD, NRD	8.56	1.31
	4	MTD, SMD	6.71	1.47	SMD, NRD	7.60	3.46	CVD, RPD	6.91	1.23
	5	SMD, NRD	5.64	1.33	CVD, NRD	5.92	2.65	MTD, SMD	5.30	1.48
	6	CVD, RPD	5.53	1.27	MTD, NRD	5.16	2.28	MTD, NRD	4.56	1.36
	7	MTD, NRD	4.87	1.39	RPD, SMD	4.77	1.93	CVD, CC	3.85	1.26
Trio	1	CVD, MTD, SMD	26.87	12.13	CVD, MTD, SMD	26.92	9.47	CVD, MTD, SMD	26.81	1.59
	2	CVD, MTD, NRD	12.60	5.48	CVD, SMD, NRD	10.06	6.39	CVD, MTD, NRD	17.66	1.51
	3	CVD, SMD, NRD	8.24	3.92	CVD, MTD, NRD	8.54	3.21	CVD, MTD, RPD	8.14	1.48
	4	CVD, RPD, SMD	6.10	3.05	CVD, RPD, SMD	6.62	2.52	CVD, MTD, CC	6.13	1.43
	5	CVD, MTD, RPD	5.53	2.49	MTD, SMD, NRD	4.97	2.49	CVD, SMD, NRD	5.98	1.49
	6	CVD, MTD, CC	3.89	2.11	CVD, SMD, MD	3.99	2.34	CVD, RPD, SMD	5.45	1.56
	7	MTD, SMD, NRD	3.53	1.86	CVD, SMD, CC	3.57	2.01	CVD, MTD, MD	4.49	1.54

**Table 3:** Most Frequent System Disease Combinations in Obese Individuals Stratified by Age Group

Comorbidity	Order	19–37			38–56			57–75			76–94		
		System Disease	%	lift	System Disease	%	lift	System Disease	%	lift	System Disease	%	lift
Singleton	1	RPD	50.00	0.00	CVD	30.30	0.65	CVD	31.12	1.07	CVD	37.55	1.04
	2	MD	20.00	0.00	SMD	17.06	0.70	SMD	22.06	1.11	SMD	27.88	1.04
	3	CVD	20.00	0.00	RPD	14.13	0.00	MTD	16.61	1.14	NRD	13.38	1.04
	4	SMD	10.00	0.00	MTD	12.02	0.70	NRD	12.27	0.91	MTD	9.76	1.14
	5	MTD	0.00	0.00	NRD	11.54	0.00	RPD	8.69	0.98	RPD	4.83	1.11
	6	NRD	0.00	0.00	MD	10.07	0.00	CC	4.95	1.04	CC	4.65	1.03
	7	CC	0.00	0.00	CC	4.87	0.00	MD	4.30	1.00	MD	1.95	1.06
Pair	1	CVD, SMD	50.00	0.00	CVD, MTD	22.91	2.19	CVD, MTD	26.77	1.54	CVD, SMD	27.04	1.34
	2	SMD, MD	50.00	0.00	CVD, SMD	10.29	1.56	CVD, SMD	14.49	1.34	CVD, MTD	23.58	1.54
	3				CVD, RPD	9.47	1.26	MTD, SMD	7.52	1.40	CVD, NRD	8.45	1.22
	4				RPD, SMD	6.17	1.76	CVD, NRD	7.14	1.22	MTD, SMD	6.28	1.40
	5				CVD, MD	5.62	1.33	SMD, NRD	6.14	1.28	SMD, NRD	6.16	1.28
	6				SMD, MD	5.08	0.00	MTD, NRD	5.66	1.31	CVD, RPD	5.22	1.23
	7				MTD, NRD	4.66	0.00	CVD, RPD	4.73	1.23	RPD, SMD	3.70	1.47
Trio	1	CVD, RPD, MD	33.33	0.00	CVD, MTD, SMD	13.26	0.00	CVD, MTD, SMD	25.90	1.57	CVD, MTD, SMD	30.99	1.27
	2	CVD, NRD, MD	33.33	0.00	CVD, MTD, NRD	11.53	0.00	CVD, MTD, NRD	13.06	1.51	CVD, MTD, NRD	12.24	1.24
	3	RPD, SMD, MD	33.33	0.00	CVD, MTD, MD	8.07	0.00	CVD, SMD, NRD	6.12	1.44	CVD, SMD, NRD	12.00	1.16
	4				CVD, MTD, RPD	7.20	0.00	CVD, RPD, SMD	5.94	1.58	CVD, RPD, SMD	6.56	1.19
	5				CVD, SMD, MD	5.19	0.00	CVD, MTD, RPD	5.71	1.47	CVD, SMD, CC	5.03	1.11
	6				CVD, RPD, SMD	4.90	0.00	MTD, SMD, NRD	4.40	1.44	CVD, MTD, RPD	4.97	1.27
	7				CVD, NRD, MD	4.03	0.00	CVD, MTD, MD	4.08	1.51	CVD, MTD, CC	4.20	1.17

**Table 4:** Characteristics of Obese Participants at Baseline, the First Follow-up, and the Last Follow-up (2002–2022)

Variables	Levels	The First Follow-up	The Last Follow-up
Age		60.23 (9.97)	70.92 (10.66)
BMI		28.06 (6.50)	26.50 (8.71)
Gender	Female	7249 (44.99)	7247 (44.97)
	Male	8865 (55.01)	8867 (55.03)
Race	White	11201 (95.67)	15392 (96.01)
	Non-white	507 (4.33)	639 (3.99)
Comorbid Disease	Cardiovascular system diseases	6460 (34.94)	9259 (25.58)
	Metabolic system disease	2142 (11.59)	7484 (20.68)
	Respiratory system disease	2361 (12.77)	3103 (8.57)
	Skeletal-muscular system diseases	4627 (25.03)	7396 (20.43)
	Neurological system diseases	679 (3.67)	4626 (12.78)
	Mental disorders	1384 (7.49)	2300 (6.35)
	Cancer	836 (4.52)	2028 (5.60)
Number of Comorbid Diseases	0	5279 (32.76)	2010 (12.47)
	1	5518 (34.24)	3299 (20.47)
	2	3560 (22.09)	4054 (25.16)
	3 or more	1757 (10.90)	6751 (41.90)

## Data Availability

The datasets analyzed during the current study are available through the UK Data Service. ELSA data can be accessed at https://beta.ukdataservice.ac.uk/datacatalogue/studies/study?id=5050. Access requires registration with the UK Data Service and agreement to the End User License terms. The data are freely available for academic research purposes.
